# Multiple virulence factors regulated by AlgU contribute to the pathogenicity of *Pseudomonas savastanoi* pv. *glycinea* in soybean

**DOI:** 10.7717/peerj.12405

**Published:** 2021-10-29

**Authors:** Viet Tru Nguyen, Nanami Sakata, Giyu Usuki, Takako Ishiga, Yoshiteru Hashimoto, Yasuhiro Ishiga

**Affiliations:** 1 Western Highlands Agriculture and Forestry Science Institute, Buon Ma Thuot, Daklak, Vietnam; 2 Faculty of Life and Environmental Sciences, University of Tsukuba, Tsukuba, Ibaraki, Japan; 3 Microbiology Research Center for Sustainability (MiCS), University of Tsukuba, Tsukuba, Ibaraki, Japan

**Keywords:** AlgU, *Pseudomonas savastanoi* pv. *glycinea*, Pathogenicity, Soybean, Coronatine, Extracellular polysaccharide

## Abstract

*Pseudomonas savastanoi* pv. *glycinea* (*Psg*) causes bacterial blight of soybean. To identify candidate virulence factors, transposon-mediated mutational analysis of *Psg* was carried out. We syringe-inoculated soybean leaves with *Psg* transposon mutants and identified 28 mutants which showed reduced virulence from 1,000 mutants screened. Next, we spray-inoculated soybean leaves with these mutants and demonstrated that the *algU* mutant showed significantly reduced virulence together with reduced bacterial populations *in planta*. Expression profiles comparison between the *Psg* wild-type (WT) and *algU* mutant in HSC broth revealed that expression of coronatine (COR)-related genes (including *cmaA* and *corR*) were down-regulated in the *algU* mutant compared with *Psg* WT. Moreover, we also showed that COR production were reduced in the *algU* mutant compared with WT. We also demonstrated that *algD*, which is related to alginate biosynthesis, showed reduced expression and biofilm formation was significantly suppressed in the *algU* mutant. Furthermore, *hrpL* also showed less expression in the *algU* mutant. These results indicate that AlgU plays a critical role in promoting *Psg* pathogenesis by regulating multiple virulence factors.

## Introduction

*Pseudomonas savastanoi* pv. *glycinea* (*Psg*) causes bacterial blight of soybean. The disease is characterized by circular necrotic lesions on leaves surrounded by a chlorotic halo (Ignjatov et al., 2007). In *P. syringae*, *P. cannabina*, and *P. savastanoi*, the phytotoxin Coronatine (COR) is important in inducing chlorosis, and contributes to bacterial growth and lesion formation (Bender et al., 1987; Budde & Ullrich, 2000; Peñaloza-Vázquez et al., 2000; Uppalapati et al., 2005; Qi et al., 2011; Sakata et al., 2021). In *Psg* PG4180, COR synthesis genes reside on a 90 kb plasmid designated p4180A (Bender, Young & Mitchell, 1991), with a 32 kb COR gene cluster which consists of two distinct regions encoding coronafacic acid (CFA) and coronamic acid (CMA) (Bender, 1999). *Psg* produces COR not only for the biological fitness of pathogens in planta (Ullrich et al., 1993) but also *in vitro* (Hoitink & Sinden, 1970; Palmer & Bender, 1993). Many studies showed the roles of COR in chlorosis, promoting lesion formation, and suspension of both stomatal and salicylic acid (SA)-dependent defenses (Peñaloza-Vázquez et al., 2000; Kloek et al., 2001; Zhao et al., 2003; Brooks, Bender & Kunkel, 2005; Melotto et al., 2006; Uppalapati et al., 2007). COR contributes to *P. syringae* pv. *tomato* (*Pst*) DC3000 virulence by suppressing the host defense response (Uppalapati et al., 2007). COR suppresses pathogen-associated molecular pattern (PAMP)-triggered immunity (PTI), especially stomatal-based defense in the early *Pst* DC3000 infection stage in *Arabidopsis thaliana* and tomato (Melotto et al., 2006; Ishiga et al., 2018).

Besides COR, the type three secretion system (T3SS) also plays a critical role in *P. syringae* virulence. The T3SS, is encoded by the *hrp* (*h*ypersensitive *r*esponse and *p*athogenicity) cluster, and transfers type three effectors (T3Es) into plant cells to suppress PTI, contribute to pathogenesis, and enhance disease symptoms and bacterial multiplication (Brooks, Bender & Kunkel, 2005; Lam et al., 2014). Furthermore, *Psg* also produces pectolytic enzymes which allow the pathogen to invade and multiply in the intercellular spaces of host tissues. This is the physiological capability of *Psg* in using plant polysaccharides and providing flexibility for its pathogen activity (Haefelet & Lindow, 1987).

AlgU, an extracytoplasmic function (ECF) sigma factor, is also important in supporting *P. syringae* growth and disease development (Markel et al., 2016). AlgU regulates between 800 to 1,000 genes (Yu et al., 2014), and importantly contributes to virulence gene regulation as well as flagellin repression (Schreiber & Desveaux, 2011; Markel et al., 2016; Bao et al., 2020). *Pst* DC3000 AlgU is not only able to regulate gene expression associated with T3SEs and the phytotoxin COR, but also alginate biosynthesis (Ishiga et al., 2018). Moreover, AlgU (previously called AlgT) also induces transcription of the *algT-mucAB* gene cluster and the *algD* operon, which are responsible for alginate biosynthesis in *Psg* PG4180 (Schenk et al., 2006). Although AlgU has been extensively studied in several *P. syringae* pathovars, its roles in *Psg* pathogenicity have not been elucidated yet.

To identify genes related to *P. syringae* pathogenicity, researchers carried out a screen for *P. syringae* mutants with reduced virulence. *Pst* DC3000 Tn*5* mutants with reduced virulence on *A*. *thaliana* found the crucial functions of COR in virulence (Brooks et al., 2004). Sakata et al. (2019) also screened for *P. cannabina* pv. *alisalensis* (*Pcal*) KB211 Tn*5* mutants with reduced virulence on cabbage plants using a dip-inoculation method, and identified multiple virulence factors including the T3SS, membrane transporters, transcription factors, and amino acid metabolism genes. Thus, it is tempting to speculate that each *P. syringae* pathovar has developed its own virulence factors. However, a screening study to identify *Psg* virulence factors has not been conducted previously.

To investigate *Psg* virulence factors, we constructed a bacterial mutant library based on transposon insertion in *Psg*, and screened for mutants with less or no chlorosis on soybean leaves after syringe-inoculation. We successfully identified several virulence factors including COR, T3Es, and AlgU. Expression profiles revealed that AlgU promotes virulence in host plants by up-regulation of COR-related gene expression. We also showed that *algU* mutant showed reduced COR production and biofilm formation compared to WT. Our results provide evidence that AlgU plays a critical role in promoting *Psg* pathogenesis.

## Materials & methods

### Bacterial strains, plasmids, and growth conditions

The bacterial strains and plasmids used in this study are listed in Table 1. *Pseudomonas* strains were routinely cultured on King’s B (KB; King, Ward & Raney, 1954) medium or mannitol-glutamate (MG; Keane, Kerr & New, 1970) medium at 28 °C. *Escherichia coli* (*E. coli*) cultures were grown on Luria-Bertani (LB; Sambrook, Fritsch & Maniatis, 1989) medium at 37 °C. The bacterial cell densities at 600 nm (OD_600_) were measured using a Biowave CO8000 Cell Density Meter (Funakoshi, Tokyo, Japan) as described in Sakata et al. (2021).

**Table 1:** Bacterial strains and plasmids used in this study.

Bacterial strain or plasmid	Locus	Relevant characteristics	Reference or source
*E. coli* strain			
DH5α		F − λ − φ80dLacZΔM15Δ (lacZYA-argF) U169 recA1 endA1 hsdR17 (rK − mK+) supE44 thi-1gyrA relA1	Takara, Kyoto, Japan
S17-1		Thi pro hsdR-hsdM + recA (chr::RP4-2-Tc::Km::Tn7)	Schäfer et al. (1994)
*P. savastanoi* pv. *glycinea*			
*Psg*		*Psg* wild-type	MAFF301684
VTB8	PsgB076_27735	*cfa6::mTn5*, Nal^r^, Km^r^	This study
VTC23	PsgB076_27795	*cmaA::mTn5*, Nal^r^, Km^r^	This study
VTD16	PsgB076_27730	*cfa5::mTn5*, Nal^r^, Km^r^	This study
VTD28	PsgB076_27725	*cfa4::mTn5*, Nal^r^, Km^r^	This study
VTD29	PsgB076_27740	*cfa7::mTn5*, Nal^r^, Km^r^	This study
VTD30	PsgB076_27720	*cfa3::mTn5*, Nal^r^, Km^r^	This study
VTE13	PsgB076_27750	*cfa8::mTn5*, Nal^r^, Km^r^	This study
VTE17	PsgB076_27740	*cfa7::mTn5*, Nal^r^, Km^r^	This study
VTF3	PsgB076_27705	*cfa2::mTn5*, Nal^r^, Km^r^	This study
VTG22	PsgB076_27735	*cfa6::mTn5*, Nal^r^, Km^r^	This study
VTG29	PsgB076_27740	*cfa7::mTn5*, Nal^r^, Km^r^	This study
VTG41	PsgB076_27725	*cfa5::mTn5*, Nal^r^, Km^r^	This study
VTH39	PsgB076_27735	*cfa6::mTn5*, Nal^r^, Km^r^	This study
VTI15	PsgB076_27740	*cfa7::mTn5*, Nal^r^, Km^r^	This study
VTR4	PsgB076_27740	*cfa7::mTn5*, Nal^r^, Km^r^	This study
VTT14	PsgB076_27805	*cmaC::mTn5*, Nal^r^, Km^r^	This study
VTT22	PsgB076_27730	*cfa5::mTn5*, Nal^r^, Km^r^	This study
VTB2	PsgB076_28870	*Type III effector protein XopAD::mTn5*, Nal^r^, Km^r^	This study
VTB52	PsgB076_09885	*Heat shock protein 90 (Hsp90)::mTn5*, Nal^r^, Km^r^	This study
VTH40	PsgB076_15537	*MFS transporter::mTn5*, Nal^r^, Km^r^	This study
VTM22	PsgB076_27425	*Hypothetical protein::mTn5*, Nal^r^, Km^r^	This study
VTO15	PsgB076_01129	*ABC transporter permease::mTn5*, Nal^r^, Km^r^	This study
VTO41	PsgB076_06035	*Sigma factor algU::mTn5*, Nal^r^, Km^r^	This study
VTP20	PsgB076_26940	*DNA-binding protein::mTn5*, Nal^r^, Km^r^	This study
VTE41	PsgB076_09720	*Unknown*, Nal^r^, Km^r^	This study
VTF6	PsgB076_10315	*Unknown*, Nal^r^, Km^r^	This study
VTO37	PsgB076_19532	*Unknown*, Nal^r^, Km^r^	This study
VTT8	PsgB076_26618	*Unknown*, Nal^r^, Km^r^	This study
*algU* mutant (VTO41) + pDSKG-*algU*		*algU* mutant complemented with pDSKG-*algU*, Nal^r^, Gen^r^	This study
Plasmid			
pBSLC1		Transposon vector constructed by ligation of pBSL118 and pHSG396 at EcoRI site, Amp^r^, Km^r^, Cm^r^	Sawada et al. (2018)
pDSK519		Broad-host-range cloning vector, Ken^r^	Keen et al. (1988)
pDSKG		Broad-host-range cloning vector, Gen^r^	This study
pDSKG-*algU*		The vector containing *algU* gene inserted into pDSKG, Gen^r^	This study

**Note:**

Amp^r^, ampicillin resistance; Cm^r^, chloramphenicol resistance; Gen^r^, gentamicin; Km^r^, kanamycin resistance; Nal^r^, nalidixic acid resistance.

### Bacterial *in vitro* growth measurements

Wild-type, the *algU* mutant, and the *algU*-complemented strain were grown at 28 °C on LB medium. The bacterial suspensions were standardized to an OD_600_ of 0.05 with LB, and bacterial growth was measured at OD_600_ for 6, 9, and 12 h.

### Plant material and inoculation procedures

Soybean plants (*Glycine max*), cultivar “Enrei”, were grown in a growth chamber at 22 °C, with approximately 60% humidity, and a supplementary light intensity of 200–350 μmol/(m^2^ s) for a 14 h photoperiod. All soybean plants used for virulence studies were 3 or 4-week-old.

**Figure 1:**
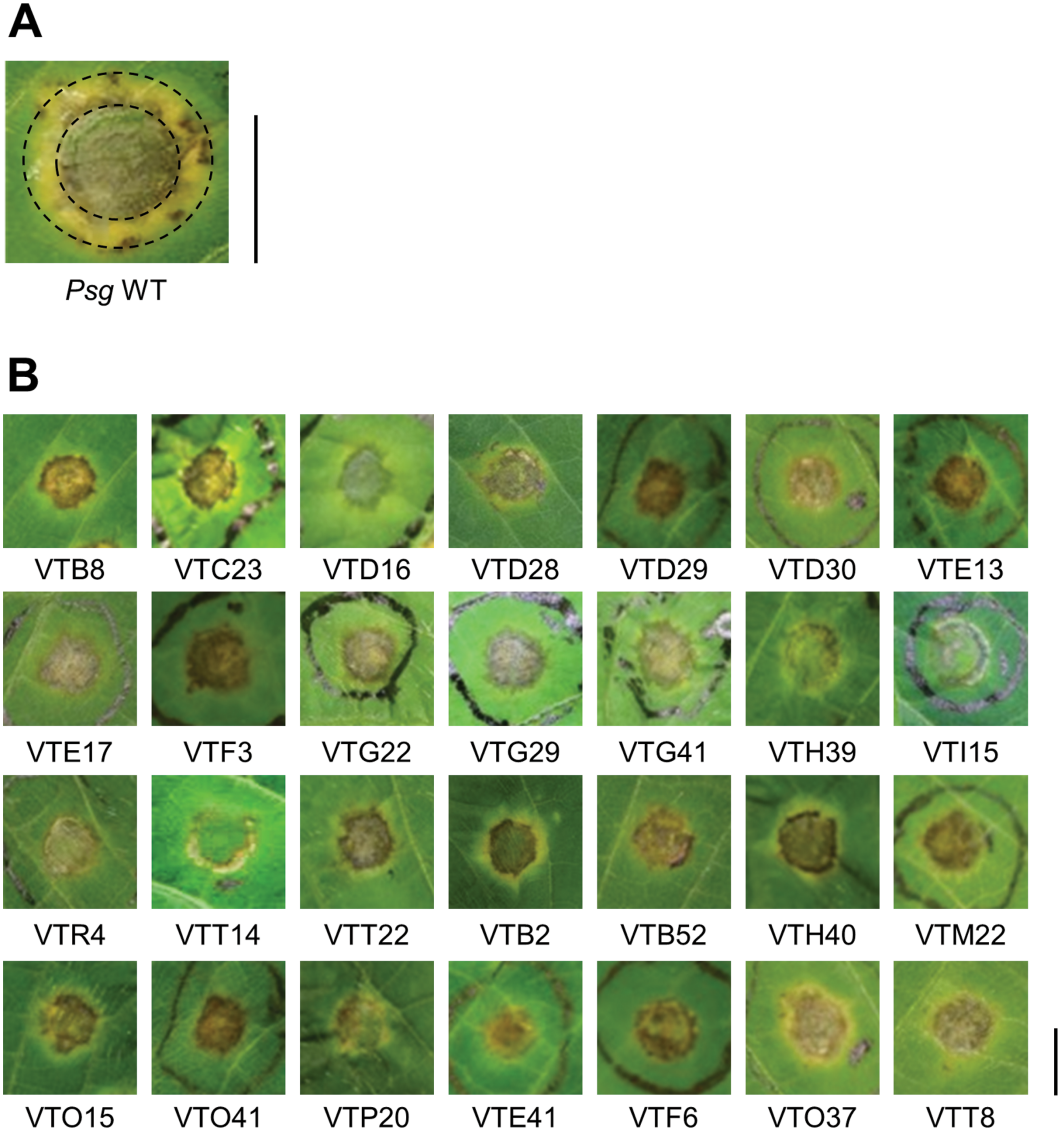
Disease symptoms on soybean leaves syringe-inoculated with *Pseudomonas savastanoi* pv. *glycinea* (*Psg*) wild-type (WT) and mutants. (A) Disease symptoms on soybean leaves syringe-inoculated with 5 × 10^7^ colony forming units (CFU)/ml of the *Psg* WT at six dpi. (B) Disease symptoms on soybean leaves syringe-inoculated with 5 × 10^7^ colony forming units (CFU)/ml of the *Psg* mutants at six dpi. Scale bars shows 1 cm.

*Psg* carrying Tn*5* transposon was syringe-infiltrated into soybean leaves at an OD_600_ of 0.1 (5 × 10^7^ CFU/ml) containing 0.02% Silwet L-77 (OSi Specialties Inc., Danbury, CT, USA). The disease symptoms were fully developed at 6 days post inoculation (dpi) (Fig. 1A). The mutants which showed different disease symptoms or virulence reduction in comparison to *Psg* WT were selected.

For spray inoculation, bacterial suspensions were applied to observe disease symptoms on whole soybean plants as described previously (Uppalapati et al., 2007). Plants were sprayed with a bacterial suspension at an OD_600_ of 0.2 (1 × 10^8^ CFU/ml) in sterile distilled water containing 0.025% Silwet L-77 until runoff. After inoculation, plants were transferred to growth chambers at 28 °C with approximately 90% to 100% humidity for 24 h in the dark before maintaining plants at approximately 70% humidity.

For syringe inoculation, bacteria were suspended at a final concentration at an OD_600_ of 0.1 (5 × 10^7^ CFU/ml), 0.01 (5 × 10^6^ CFU/ml), and 0.001 (5 × 10^5^ CFU/ml), and infiltrated with a one-ml blunt syringe into leaves. The plants were then incubated at 70–80% humidity for the rest of the experimental period. Leaves were removed and photographed at five dpi.

To measure bacterial growth in soybean leaves after spray inoculation, individual second leaf pairs were selected at six dpi, weighed and surface-sterilized in 5% H_2_O_2_ for 3 min, and then rinsed three times with sterile water. The leaves were then homogenized, and appropriate dilutions were plated on KB medium containing the appropriate antibiotics. The bacterial colony forming units (CFU) were normalized as CFU/g using the total inoculated leaf mass. The population at 0 dpi was estimated using leaves harvested 1 h post inoculation (hpi) without surface-sterilization. For syringe-inoculation, leaf discs were harvested using a 3.5 mm-diameter cork-borer from syringe-infiltrated leaf zone. The leaves were then homogenized, and appropriate dilutions were plated on KB medium containing the appropriate antibiotics. The bacterial colony forming units (CFU) were normalized as CFU/cm^2^ using the leaf square meters. The bacterial populations were evaluated in at least three independent experiments.

### Transposon-mediated mutagenesis and identification of mutated genes

Transposon-mediated mutagenesis was carried out, as described previously (Sakata et al., 2019). Briefly, pBSLC1 (Sawada et al., 2018) carrying mini-Tn*5* transposon were transferred into *Psg* to build a mutant library. We developed more than 1,000 individual *Psg* mutant lines. After the inoculation assay, we identified the mutated genes by rescuing the transposon insertion sites into an *E. coli* plasmid and sequencing (Sakata et al., 2019).

### Complementation of the *algU* mutant

The *algU*-complemented strain was constructed as described in Ishiga et al. (2018). Briefly, the pDSKG vector was made from pDSK519 vector (Keen et al., 1988) by replacing kanamycin cassette to gentamycin. The *algU* and promoter region were transferred into the pDSKG vector to generate pDSKG-*algU*. The pDSKG-*algU* construct was introduced into the *algU* mutant by electrophoresis to generate the complemented strain.

### Real-time quantitative RT-PCR

For *Psg* gene expression profiles, data were collected as previously described in Sakata et al. (2021). Specifically, bacteria were grown in HS medium optimized for COR production (HSC; Palmer & Bender, 1993) for 3 and 48 h. Bacterial RNA was extracted using the ReliaPrep RNA Cell Miniprep System Kit (Promega, WI, USA) according to the manufacture’s protocol. Two micrograms of total RNA were treated with gDNA Remover (TOYOBO, Osaka, Japan) to eliminate genomic DNA, and the DNase-treated RNA was reverse transcribed using the ReverTra Ace qPCR RT Master Mix (TOYOBO). The cDNA (1:10) was then used for RT-qPCR using the primers shown in Table S1 with THUNDERBIRD SYBR qPCR Mix (TOYOBO) on a Thermal Cycler Dice Real Time System (TaKaRa). *Psg outer membrane lipoprotein I* (*oprI*) was used to normalize gene expression.

### COR quantification by HPLC

*Psg* WT, the *algU* mutant, and the *algU*-complemented strain were cultured in HSC for 7 days. Culture supernatant was obtained by centrifugation (12,000 × g for 5 min). Cell pellets were dried at 65 °C and weighed. The 500 µl of supernatants were extracted twice with 500 µl of ethyl acetate and 25 µl of HCl, and the organic phase was transferred to a new microcentrifuge tube. The sample was dried by centrifugal evaporator at 55 °C, and the dried sample was dissolved with 0.05% trifluoroacetic acid (TFA)/acetonitrile (9:1, v/v). The culture supernatant was analyzed by HPLC with a Shimadzu LC20A system equipped with a Symmetry C8 column (4.6 × 250 mm; Waters Corporation, MA, USA) as described previously (Sakata et al., 2021).

### Hypertrophy-inducing activity assay on potato tuber tissue

Potato tubers were cut from the central tuber portion to ensure samples of high uniformity. After washing in tap water for 5 min, each disc was washed with sterile distilled water several times. Potato tuber discs were inoculated using toothpicks by placing the tip in *Psg* WT, COR-defective mutants (*cfa6* and *cmaA*), the *algU* mutant, and the *algU*-complemented strain on a KB medium plate, and then placing the toothpick on the potato tuber disc. The discs were then placed at 23 °C incubator (darkness) for 5 days. Photographs were taken at five dpi.

### Biofilm formation assay

Biofilm formation was assayed as described previously (Shao et al., 2019). Briefly, the bacterial strains were incubated overnight in LB broth and resuspended in fresh LB broth to an OD_600_ of 0.1. Bacterial suspensions (120 μl) were put into 96-well plates and incubated at 28 °C for 24, 48, 72, and 96 h. The bacterial solutions were discarded and washed three times with distilled water. The biofilm forming bacteria were treated with 150 μl of 0.1% crystal violet (CV; Fujifilm, Tokyo, Japan) for 20 min without shaking. The dye was discarded and washed twice with distilled water. The plate was dried completely, subsequently the biofilm was eluted with 150 μl of 100% ethanol, and the CV were dissolved completely. Finally, the eluted biofilm sample’s absorbance was measured at OD_595_.

## Results

### Identification and characterization of reduced virulence mutants

To identify *Psg* virulence genes, we screened 1,000 transposon insertion mutants for reduced disease symptoms on soybean leaves using the syringe-infiltration method. Disease symptoms caused by *Psg* WT showed a small water-soaked lesion surrounded by regions of chlorosis (Fig. 1A). A total of 28 mutants showed no or less chlorosis at six dpi (Fig. 1B). Seventeen mutants out of 28 had transposon insertions in genes encoding COR biosynthesis-related genes (Table 1). Soybean leaves inoculated with COR biosynthesis mutants (VTD29, VTE13, VTI15, and VTR4), the *algU* mutant (VTO41), and an unknown-function mutant (VTF6) showed no chlorosis (Fig. 1B).

### Reduced disease symptoms and bacterial growth in soybean

**Figure 2:**
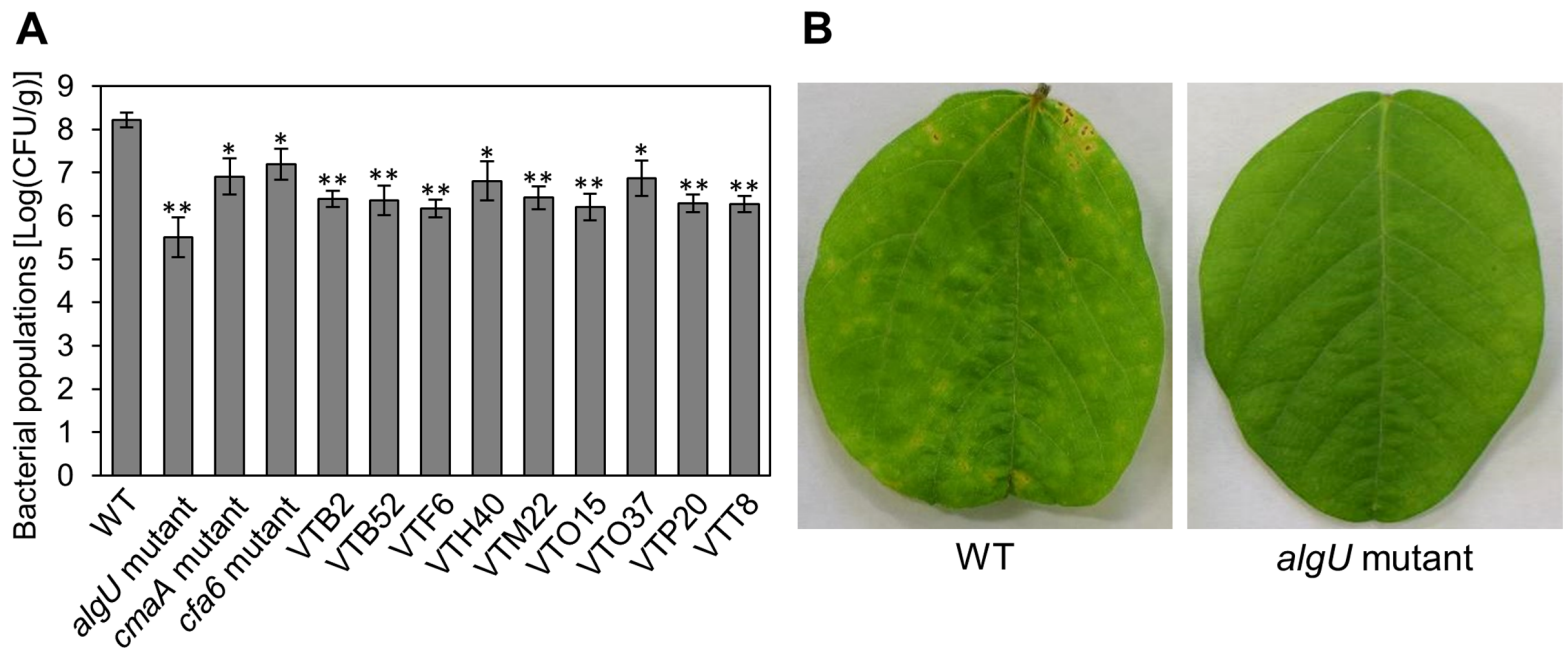
Bacterial populations and disease symptoms in soybean leaves. (A) Bacterial populations in leaves spray-inoculated with *Pseudomonas savastanoi* pv. *glycinea* (*Psg*) wild-type (WT) and mutants (1 × 10^8^ colony forming units (CFU)/ml). Bacterial populations in leaves were estimated at 6 days post inoculation (dpi) by dilution plating on selective medium as described in the methods. Vertical bars indicate standard error for three independent experiments. Asterisks indicate a significant difference from WT in a *t* test (*P < 0.05; **P < 0.01). (B) Disease symptoms in leaves spray-inoculated with the *Psg* WT and the *algU* mutant (1 × 10^8^ colony forming units (CFU)/ml) at six dpi.

We identified the 28 mutants which showed reduced disease symptoms compared to *Psg* WT by syringe-inoculation (Fig. 1B). We further investigated whether these mutants also showed reduced virulence *via* spray-inoculation, and selected two mutants related to COR biosynthesis (*cmaA* and *cfa6*), and others. Soybean leaves inoculated with all mutants showed significantly reduced bacterial populations than those of *Psg* WT (Fig. 2A). Among all mutants, the *algU* mutant (VTO41) showed dramatically reduced bacterial populations and disease symptoms at six dpi (Figs. 2A and 2B). To confirm whether the altered *algU* mutant phenotype originates from a corresponding mutation, an *algU*-complemented strain was generated. *Psg* WT and the *algU*-complemented strain showed the same bacterial population levels as well as disease symptom development in soybean (Figs. S1A and S1B). We also confirmed that *algU* is apparently dispensable for *Psg* growth in rich LB medium, since no growth difference was observed among WT, *algU* mutant, and *algU*-complemented strain (Fig. S2).

**Figure 3:**
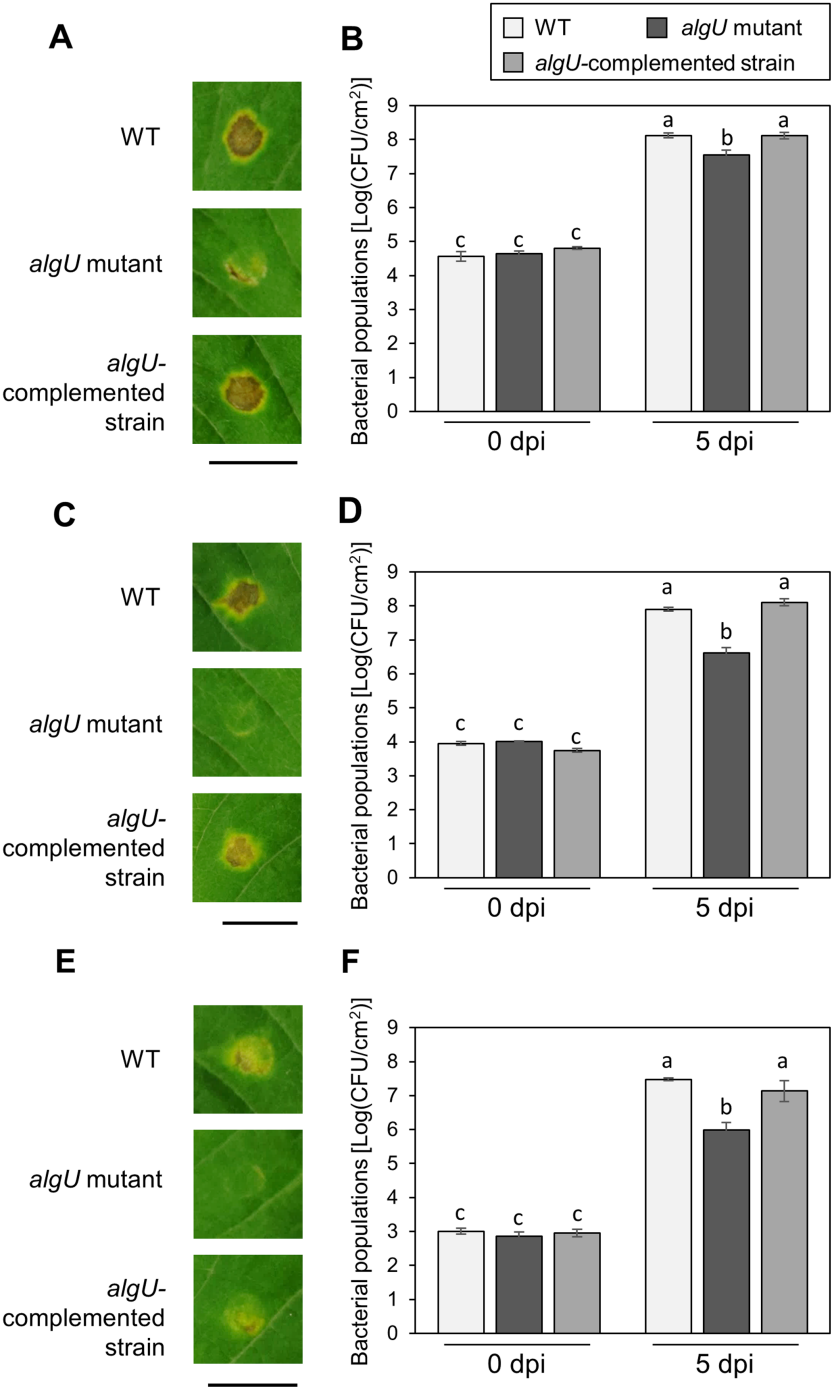
Disease symptoms and bacterial populations in soybean leaves after syringe inoculation. Disease symptom and bacterial populations in leaves syringe-inoculated with *Pseudomonas syringae* pv. *glycinea* (*Psg*) wild-type (WT), the *algU* mutant, and the *algU*-complemented strain at 5 × 10^7^ colony forming units (CFU)/ml (A, B), 5 × 10^6^ CFU/ml (C, D), and 5 × 10^5^ CFU/ml (E, F), respectively. Photographs were taken at 5 days post inoculation (dpi). Scale bars shows 1 cm. Bacterial populations in leaves were estimated at five dpi by dilution plating on selective medium as described in the methods. Vertical bars indicate standard error for three independent experiments. The different letters indicate significant statistical differences (*P* < 0.05, Turkey’s HSD test).

To further investigate the algU contribution to *Psg* virulence, we conducted syringe infiltration with WT, *algU* mutant, and *algU*-complemented strain. As a result, the *algU* mutant showed reduced symptoms and bacterial populations at all inoculum levels we tested (Figs. 3A–3F). Taken together, these results indicate that AlgU contributes to growth both on leaf surface and in apoplast, and to causing disease.

### AlgU regulates the expression of *Psg* virulence genes in HSC medium

**Figure 4:**
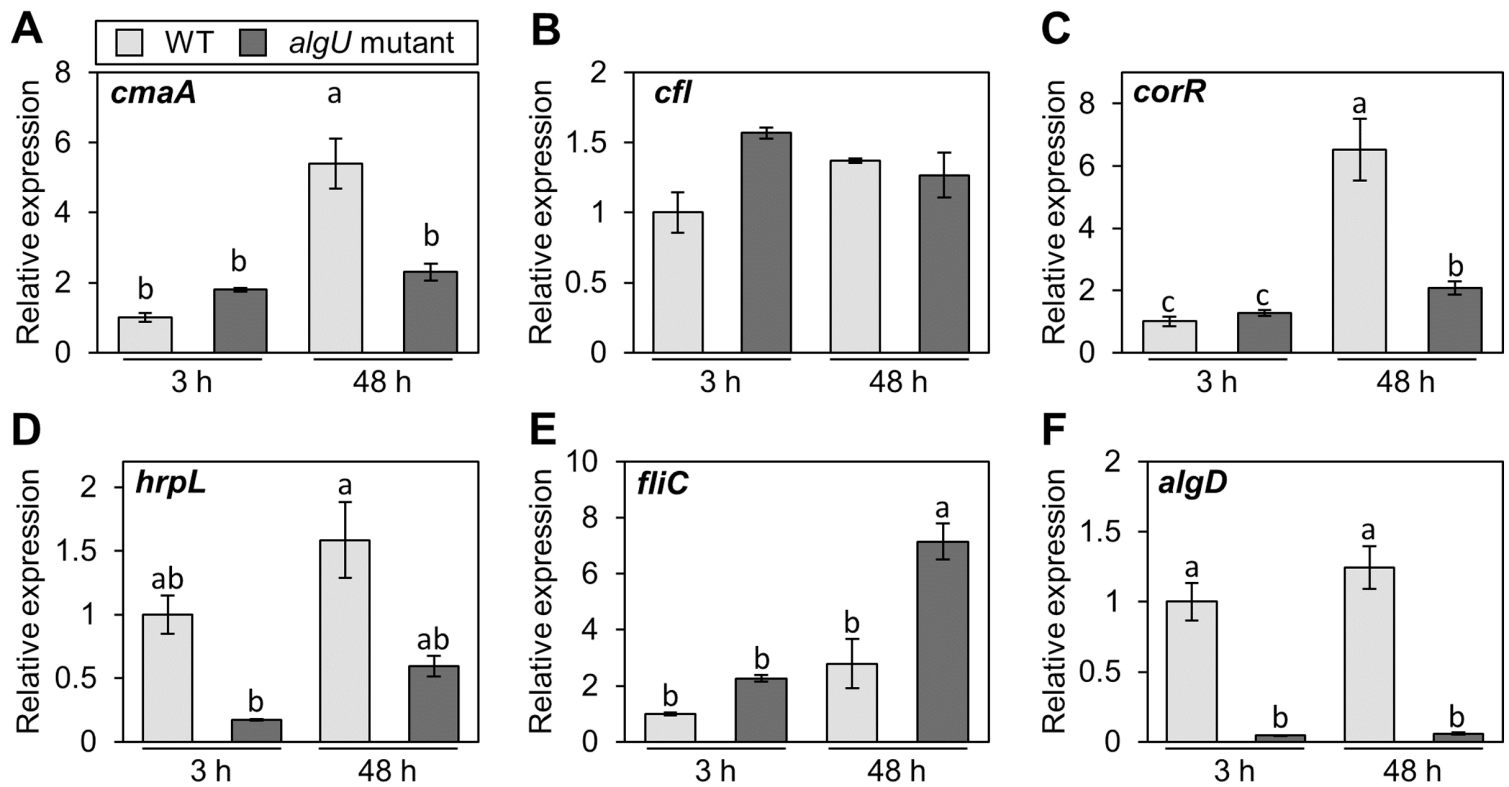
Gene expression profiles involved in the virulence of *Pseudomonas savastanoi* pv. *glycinea* (*Psg*) wild-type (WT) and *algU* mutant in liquid HSC broth. *Psg* WT and *algU* mutant were grown in HSC broth for 3 and 48 h, adjusted to an OD_600_ of 0.1, and grown again in fresh HSC broth for 3 h. Gene expression was normalized using the housekeeping gene *Psg outer membrane lipoprotein I* (*oprI*) by real-time quantitative reverse transcription-polymerase chain reaction (RT-qPCR) with gene-specific primer sets (Table S1). (A) *cmaA*, (B) *cfl*, (C) *corR*, (D) *hrpL*, (E) *fliC*, and (F) *algD*. Vertical bars indicate the standard error for three biological replicates. The different letters (a–c) indicate a significantly statistical difference (*P* < 0.05, Turkey’s HSD test).

*Pst* DC3000 AlgU positively regulates virulence gene transcription (Markel et al., 2016; Ishiga et al., 2018). To investigate whether *Psg* AlgU also regulates virulence genes, we analyzed virulence gene expression profiles in HSC medium. COR biosynthesis-related genes including *cmaA* and *corR*, in the *algU* mutant showed reduced expression at 48 h after incubation (Figs. 4A and 4C). Moreover, *hrpL*, encoding HrpL (an alternative sigma factor recognizing the *hrp* box in the promoter of T3SS genes), also showed significantly less expression in the *algU* mutant at both 3 and 48 h after incubation compared to *Psg* WT (Fig. 4D). These results indicate that AlgU positively regulates COR biosynthesis-related genes and *hrpL*.

To investigate whether AlgU can coordinate gene expression involved in *Psg* motility, we determined the expression profile of *fliC* (encoding flagellin, relating to flagellar mobility). At 3 h after incubation, there was no difference in the flagellar-encoding gene expression between the *algU* mutant and *Psg* WT. However, after 48 h incubation, relative *fliC* expression was greater in the *algU* mutant compared to *Psg* WT (Fig. 4E). Additionally, *algD* expression was down regulated in the *algU* mutant (Fig. 4F), indicating that AlgU positively regulates alginate biosynthesis-related genes.

### AlgU contributes to COR biosynthesis and biofilm formation in *Psg*

**Figure 5:**
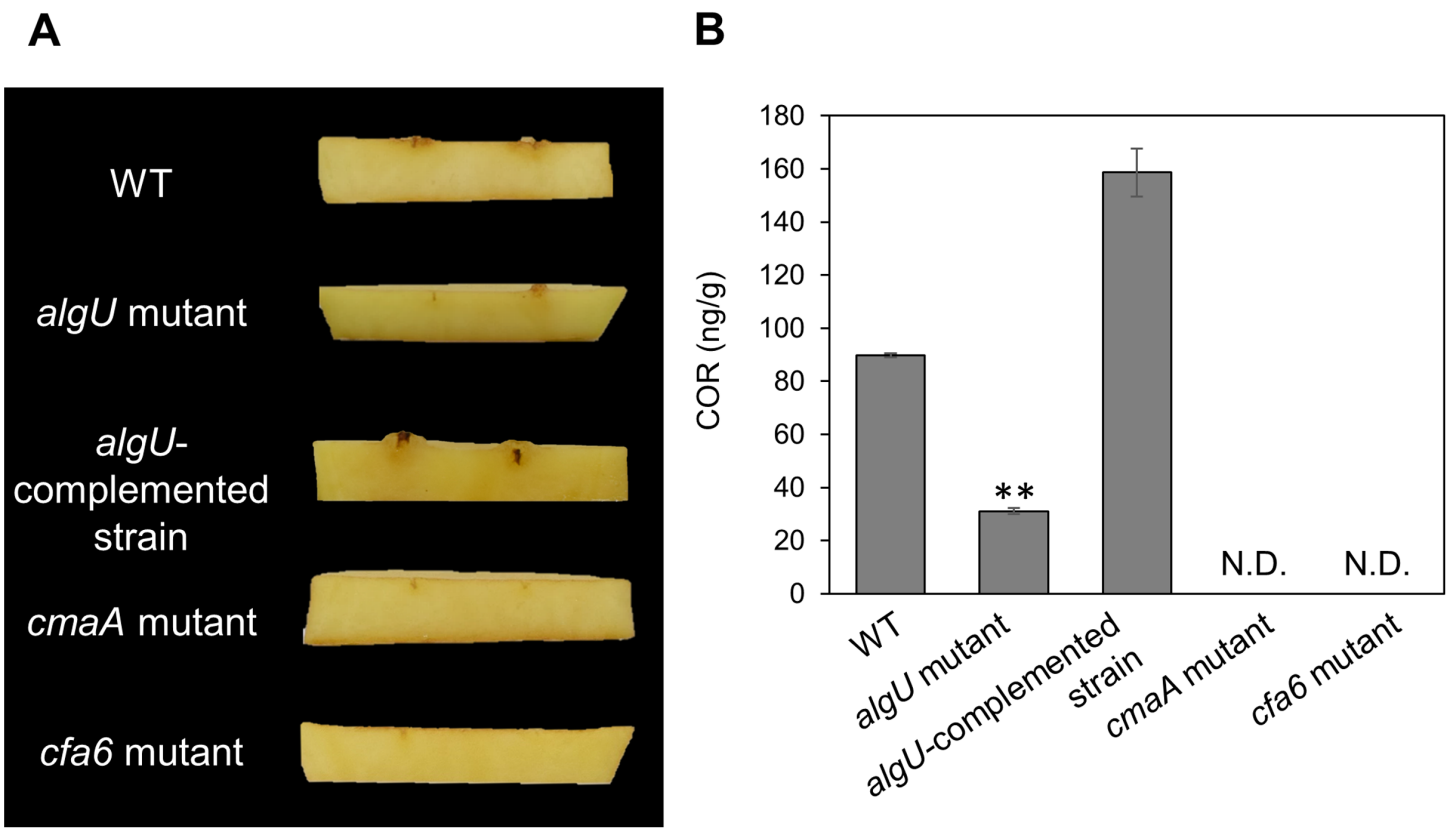
COR quantification of *Pseudomonas savastanoi* pv. *glycinea* (*Psg*) wild-type (WT), the *algU* mutant, and the *algU*-complemented strain. (A) Observation of hypertrophy-inducting activity on potato tuber tissue inoculated with *Pseudomonas savastanoi* pv. *glycinea* (*Psg*) wild-type (WT), COR-defective mutants (*cmaA* and *cfa6*), the *algU* mutant, and the *algU*-complemented strain. Potato tuber discs were inoculated using toothpicks by placing the tips in the *Psg* WT, *cmaA*, *cfa6*, *algU* mutant, and the *algU*-complemented strain on a KB medium plate and then placing the toothpick on the potato tuber disc. Photographs were taken at five dpi. (B) COR quantification of *Pseudomonas savastanoi* pv. *glycinea* (*Psg*) wild-type (WT) and the *algU* mutant grown in liquid HS broth by HPLC. *Psg* WT, the *algU* mutant, and the *algU*-complemented strain were cultured in HSC broth for 7 days. HPLC analysis was conducted by a Shimadzu LC20A system equipped with a Symmetry C8 column. COR in the culture supernatant was identified, as compared with authentic COR as the standard. Vertical bars indicate the standard error for three biological replicates. Asterisks indicate a significant difference from WT in a *t* test (**P < 0.01). N.D. indicates not detected by HPLC.

We demonstrated that COR biosynthesis-related genes in the *algU* mutant showed reduced expression in HSC medium (Figs. 4A–4D). To investigate whether AlgU contributes to COR production, we first conducted a hypertrophy-inducting activity test on potato tuber tissues for COR detection (Sakai et al., 1979; Völksch, Bublitz & Fritsche, 1989). Potato tuber tissues inoculated with *Psg* WT showed hypertrophy response, but those inoculated with COR-defective mutants (*cmaA* and *cfa6*) showed no response (Fig. 5A). The *algU* mutant-inoculated potato tuber tissues showed less hypertrophy response compared to those inoculated with *Psg* WT and the reduction was restored in the *algU*-complemented strain (Fig. 5A). Furthermore, we also quantified COR production by using HPLC. *Psg* WT produced around 90 ng/g of COR in HSC medium (Fig. 5B). However, the *algU* mutant produced only around one third as much COR compared to *Psg* WT (Fig. 5B). We also confirmed that the *algU*-complemented strain recovered COR production more than WT (Fig. 5B). Taken together, these results suggest that AlgU contributes to COR biosynthesis in *Psg*.

**Figure 6:**
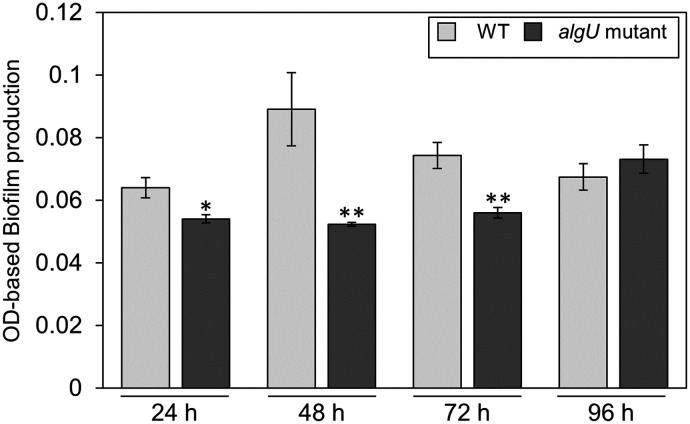
Biofilm biomass measurement of *Pseudomonas savastanoi* pv. *glycinea* (*Psg*) wild-type (WT) and the *algU* mutant with crystal violet (CV) grown in LB broth at 28 °C for 24, 48, 72, and 96 h. The biofilm eluted sample’s absorbance was measured at OD_595_. Vertical bars indicate the standard error for three biological replicates. Asterisks indicate a significant difference from WT in a *t* test (*P < 0.05, **P < 0.01).

Since we also demonstrated that alginate biosynthesis-related genes *algD* showed reduced expression in *algU* mutant (Fig. 4F), we next investigated the biofilm formation ability in *Psg* WT and the *algU* mutant. The *algU* mutant showed a reduction in biofilm formation at 24, 48, and 72 hpi (Fig. 6). These results suggest that AlgU also contributes to biofilm formation in *Psg*.

## Discussion

We attempted to identify *Psg* virulence factors that are crucial in soybean pathogenicity. We screened 1,000 *Psg* mutants by syringe-infiltration and identified 28 mutants with reduced virulence (Fig. 1B). Several important virulence factors contribute to *Psg* virulence including COR, the T3SS, and AlgU (Table 1). Sigma factor AlgU regulates not only *algD*, but also other virulence genes including *hrpL* and COR (Figs. 4A–4F). Our study provides new insights into AlgU function as a global regulatory hub for *Psg* pathogenicity by regulating the expression of multiple virulence genes.

Our screening identified that 17 out of 28 reduced virulence mutants were related to COR biosynthesis genes. These COR biosynthesis mutants were mostly disrupted by Tn*5* on the *cfa* and *cma* operons (Table 1). The *cfa* and *cma* operons encode enzymes related to CFA and CMA biosynthesis, respectively, the two elements that are ligated together to form COR (Bender, 1999). Together, these results indicate that COR is an important *Psg* virulence factor.

The *algU* mutant showed reduced virulence in plants both spray-and syringe-inoculated (Figs. 2, S1, 3), indicating that AlgU contributes to *Psg* multiplication both on leaf surface and in apoplast, and causing disease. Our results indicate that AlgU regulate several virulence factors. Firstly, gene expression related to COR biosynthesis, such as *cmaA* and *corR* (but not *cfl*) were suppressed in the *algU* mutant (Figs. 4A–4C). Moreover, COR production in the *algU* mutant also less than that of *Psg* WT (Figs. 5A and 5B), suggesting that AlgU contributes to COR production in *Psg*. Consistent with our results, Ishiga et al. (2018) demonstrated that gene expression related to COR biosynthesis was suppressed during *Pst* DC3000 *algU* mutant infection. Furthermore, AlgU also contributes to *Pst* DC3000 virulence by regulating COR production to overcome stomatal-based defense (Ishiga et al., 2018). Together, these results suggest that AlgU suppresses stomatal-based defense in the early *Psg* infection stage with soybean plants. Moreover, COR contributes to virulence by overcoming apoplastic defense as well as stomatal-based defense in *Pcal* (Sakata et al., 2021). Further study on COR contribution in *Psg* virulence will be needed to understand AlgU-mediated COR regulation.

Secondary, the *algD* expression profile in the *algU* mutant was significantly reduced in comparison with *Psg* WT, indicating that AlgU is important in regulating alginate biosynthesis gene expression (Fig. 4F). This result was consistent with a previous report in *Pst* DC3000 (Ishiga et al., 2018), in which *algD* expression was significantly suppressed in an *algU* mutant. Further, alginate plays a crucial role in epiphytic fitness and survival, and contributes to *P. syringae* virulence (Yu et al., 1999). Alginate is one of the exopolysaccharides (EPSs), which are the major components of biofilms, in *Psg* (Osman, Fett & Fishman, 1986; Sutherland, 2001). We demonstrated that biofilm formation was significantly decreased in the *algU* mutant compared with *Psg* WT (Fig. 6). *Psg* PG4180 AlgU is important in virulence and bacterial growth in host plants, but it is not dependent on alginate production (Schenk, Weingart & Ullrich, 2008; Yu et al., 2014). Additionally, AlgU, but not AlgD plays a crucial role in *Pst* DC3000 virulence (Markel et al., 2016). Together, it is tempting to speculate that alginate function in virulence differs in each *P. syringae* pathovar. Thus, further study is needed to understand AlgU regulating genes involved in biofilm formation and alginate function in *Psg* virulence.

Thirdly, expression profiles also revealed *hrpL* transcripts were suppressed in the *algU* mutant compared with *Psg* WT (Fig. 4D). *Pst* DC3000 AlgU functions to regulate *hrpL* expression (Ishiga et al., 2018). Markel et al. (2016) also demonstrated that AlgU plays an important role in virulence by regulating the expression of T3Es and *hrpL*. In *P. syringae*, both the T3SS and T3Es genes are in turn encoded by the *hrp* gene cluster, while the sigma factor HrpL directly regulates both *hrc* and *hop* genes (Lam et al., 2014). Although many studies were caried out to elucidate the functions and mode of actions of the T3SS and its T3Es, AlgU regulation on the T3SS system is still unknown in *P. syringae* infection processes. Therefore, further precise characterization of AlgU-mediated T3SS regulation will be needed to understand global gene expression networks during *Psg* infection.

Lastly, *fliC* transcripts in the *algU* mutant were increased compared with those of *Psg* WT (Fig. 4E). In *Pst* DC3000, AlgU not only downregulates flagellar and chemotaxis genes *in vitro* (Markel et al., 2016), but also negatively regulates *fliC* expression during infection (Bao et al., 2020). *fliC* encodes the flagellin protein including the flg22 epitope which triggers PTI (Felix et al., 1999; Zipfel et al., 2004; Parys et al., 2021; Colaianni et al., 2021). Recent studies reported the important role of AlgU in de-flagellation during the *P. syringae*-plant interaction to reduce PTI activation, and promote bacterial fitness in its host (Bao et al., 2020). Likewise, AlgU also plays an important role in de-flagellation of *P. syringae* pv. *maculicula* ES4236, in which transposon inactivation of AlgW led to decreased AlgU activity and increased the flagella expression, as well as reduced bacterial growth *in planta* (Schreiber & Desveaux, 2011). Therefore, it is tempting to speculate that high levels of flagellin protein production in the *algU* mutant activate PTI.

## Conclusions

Our findings indicate that multiple virulence factors regulated by AlgU, including COR biosynthesis, biofilm formation, and T3SS, contributes to *Psg* virulence in soybean. Our findings help to expand understanding of AlgU roles in *Psg* virulence. Further studies on AlgU regulated mechanisms will be needed to fully understand *Psg* virulence.

## Supplemental Information

10.7717/peerj.12405/supp-1 Supplemental Information 1 Datasets used in this study.

10.7717/peerj.12405/supp-2 Supplemental Information 2 Disease symptoms and bacterial population dynamics in soybean leaves spray-inoculated with 1 × 10^8^ colony forming units (CFU)/ml of the *Pseudomonas savastanoi* pv. *glycinea* (*Psg*) wild-type (WT), *algU* mutant, and *algU*. (A) Disease symptoms in leaves spray-inoculated with 1 × 10^8^ colony forming units (CFU)/ml of the *Psg* WT, *algU* mutant, and *algU*-complemented line (*algU* mutant + pDSKG-*algU*) at six dpi. (B) Bacterial populations in leaves were estimated at 2, 4, and 6 dpi by dilution plating on selective medium as described in the methods. Vertical bars indicate the standard error for three independent experiments. The different letters indicate significant statistical differences (*P* < 0.05, Turkey’s HSD test).

10.7717/peerj.12405/supp-3 Supplemental Information 3 *Pseudomonas savastanoi* pv.*glycinea* WT, the *algU* mutant and the *algU*-complemented line growth in LB medium. All strains were adjusted to an OD_600_ of 0.05 in LB medium and incubated with shaking at 28 °C.Bacterial growth was quantified at 6, 9, and 12 h. Vertical bars indicate the standard error for three biological replicates. Different letters indicate a significant difference among treatments based on a Tukey’s HSD test (*P* < 0.05).

10.7717/peerj.12405/supp-4 Supplemental Information 4 Primers used in this study.

## Additional Information and Declarations

### Data Availability

The following information was supplied regarding data availability: The raw measurements are available in the Supplementary File.
