# Supplementary figures and images for: Multiple virulence factors regulated by AlgU contribute to the pathogenicity of Pseudomonas savastanoi pv. glycinea in soybean
Source: PeerJ. 2021 Oct 29;9:e12405. doi: 10.7717/peerj.12405 (PMC8559602; doi:10.7717/peerj.12405)

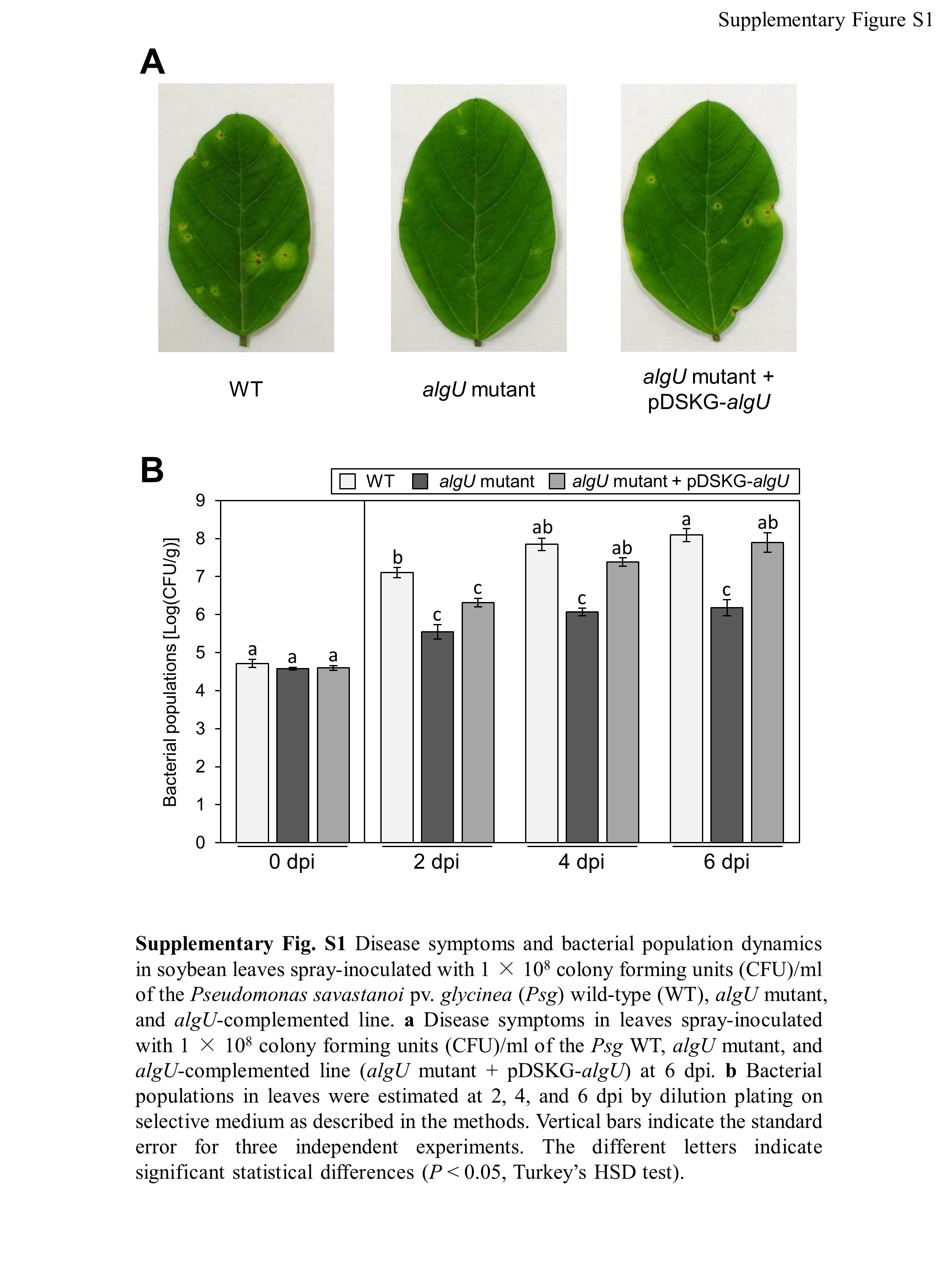

Supplement: Supplemental Information 2 — (A) Disease symptoms in leaves spray-inoculated with 1 × 10^8^ colony forming units (CFU)/ml of the Psg WT, algU mutant, and algU-complemented line (algU mutant + pDSKG-algU) at six dpi. (B) Bacterial populations in leaves were estimated at 2, 4, and 6 dpi by dilution plating on selective medium as described in the methods. Vertical bars indicate the standard error for three independent experiments. The different letters indicate significant statistical differences (P < 0.05, Turkey’s HSD test). [file peerj-09-12405-s002.png]

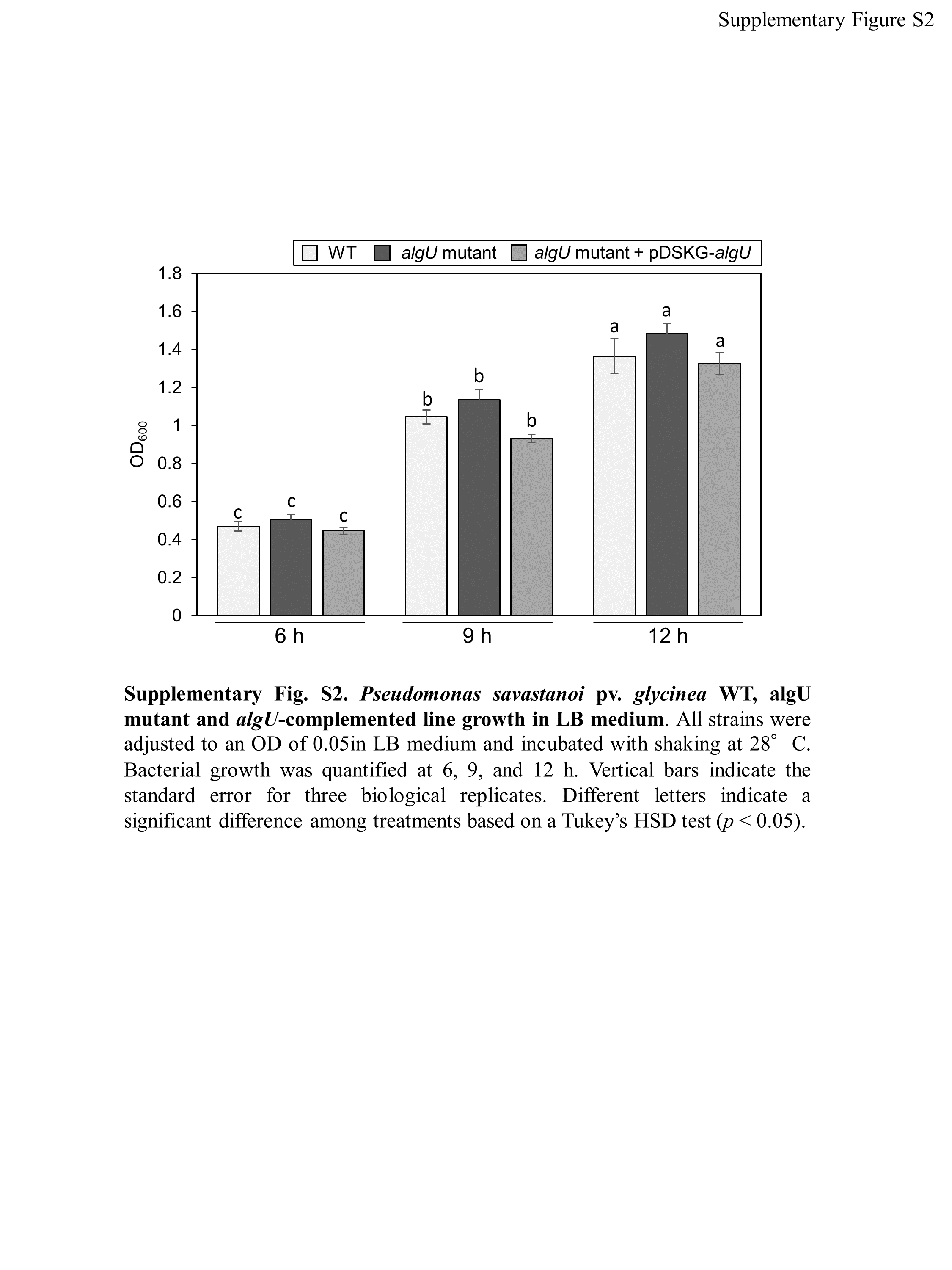

Supplement: Supplemental Information 3 — All strains were adjusted to an OD600 of 0.05 in LB medium and incubated with shaking at 28 °C.Bacterial growth was quantified at 6, 9, and 12 h. Vertical bars indicate the standard error for three biological replicates. Different letters indicate a significant difference among treatments based on a Tukey’s HSD test (P < 0.05). [file peerj-09-12405-s003.png]
