# Supplementary material for: Multiple virulence factors regulated by AlgU contribute to the pathogenicity of Pseudomonas savastanoi pv. glycinea in soybean
Source: PeerJ. 2021 Oct 29;9:e12405. doi: 10.7717/peerj.12405 (PMC8559602; doi:10.7717/peerj.12405)
Supplement: Supplemental Information 4 [file peerj-09-12405-s004.docx]

| Supplementary Table S1 Primers used in this study | | | |
| --- | --- | --- | --- |
| Gene | Primer name | Primer usage | Primer sequence |
| *cmaA* | cmaA-FW | Expression analysis on *Psg* | AAAGCCTACCGCCGATTT |
| *cmaA* | cmaA-RV | Expression analysis on *Psg* | CGTCTGGAGCTGTTGATAAGT |
| *cfl* | cfl-FW | Expression analysis on *Psg* | GAACTGGTGGCGTTGTACTAT |
| *cfl* | cfl-RV | Expression analysis on *Psg* | GTGGAGCAGATGCTCAATTTC |
| *corR* | corR-FW | Expression analysis on *Psg* | GGGATTGACGAACAAGGAGAT |
| *corR* | corR-RV | Expression analysis on *Psg* | GACGTCTGGAGGATAGTCAGTA |
| *hrpL* | hrpL-FW | Expression analysis on *Psg* | AGACCTGGTTGTGTGGTATTG |
| *hrpL* | hrpL-RV | Expression analysis on *Psg* | CCCGTCTACCTGATGAGTGATA |
| *algD* | algD-FW | Expression analysis on *Psg* | CATGCCAGTAAACCGCAAAC |
| *algD* | algD-RV | Expression analysis on *Psg* | GACGATACGTGCGGAATCTT |
| *fliC* | fliC-FW | Expression analysis on *Psg* | CTTCGAACCAGATCACTCTGAC |
| *fliC* | fliC-RV | Expression analysis on *Psg* | GTCTCTGCAGTGGTGCTATC |
| *oprI* | oprI-FW | Expression analysis on *Psg* | GCAGCAGCGTATCCAAAGA |
| *oprI* | oprI-RV | Expression analysis on *Psg* | TGGCTTCGTCAGCAGTTT |
